# Multimodal functional and structural neuroimaging investigation of major depressive disorder following treatment with duloxetine

**DOI:** 10.1186/s12888-015-0457-2

**Published:** 2015-04-14

**Authors:** Cynthia HY Fu, Sergi G Costafreda, Anjali Sankar, Tracey M Adams, Mark M Rasenick, Peng Liu, Robert Donati, Luigi A Maglanoc, Paul Horton, Lauren B Marangell

**Affiliations:** School of Psychology, University of East London, Arthur Edwards Building, Rm 3.11, Water Lane, London, E15 4LZ UK; Centre for Affective Disorders, Institute of Psychiatry, Psychology and Neuroscience (IoPPN), King’s College London, London, UK; Department of Psychiatry, University College London, London, UK; Department of Old Age Psychiatry, IoPPN, King’s College London, London, UK; Psychological Services, Warneford Hospital, Oxford, UK; University of Illinois at Chicago, College of Medicine, Chicago, IL USA; Jesse Brown VA Medical Center, Chicago, IL USA; Eli Lilly and Company, Indianapolis, IN USA; Illinois College of Optometry, Chicago, IL USA; Department of Neurodegenerative Disease, Institute of Neurology, University College London, London, UK; University of Texas Health Science Center, Houston, TX USA

**Keywords:** Antidepressant, BOLD, Brain, Function, Prognosis, Predictor, Structure

## Abstract

**Background:**

Longitudinal neuroimaging studies of major depressive disorder (MDD) have most commonly assessed the effects of antidepressants from the serotonin reuptake inhibitor class and usually reporting a single measure. Multimodal neuroimaging assessments were acquired from MDD patients during an acute depressive episode with serial measures during a 12-week treatment with the serotonin-norepinephrine reuptake inhibitor (SNRI) duloxetine.

**Methods:**

Participants were medication-free MDD patients (n = 32; mean age 40.2 years) in an acute depressive episode and healthy controls matched for age, gender, and IQ (n = 25; mean age 38.8 years). MDD patients received treatment with duloxetine 60 mg daily for 12 weeks with an optional dose increase to 120 mg daily after 8 weeks. All participants had serial imaging at weeks 0, 1, 8, and 12 on a 3 Tesla magnetic resonance imaging (MRI) scanner. Neuroimaging tasks included emotional facial processing, negative attentional bias (emotional Stroop), resting state functional MRI and structural MRI.

**Results:**

A significant group by time interaction was identified in the anterior default mode network in which MDD patients showed increased connectivity with treatment, while there were no significant changes in healthy participants. In the emotional Stroop task, increased posterior cingulate activation in MDD patients normalized following treatment. No significant group by time effects were observed for happy or sad facial processing, including in amygdala responsiveness, or in regional cerebral volumes. Reduced baseline resting state connectivity within the orbitofrontal component of the default mode network was predictive of clinical response. An early increase in hippocampal volume was predictive of clinical response.

**Conclusions:**

Baseline resting state functional connectivity was predictive of subsequent clinical response. Complementary effects of treatment were observed from the functional neuroimaging correlates of affective facial expressions, negative attentional bias, and resting state. No significant effects were observed in affective facial processing, while the interaction effect in negative attentional bias and individual group effects in resting state connectivity could be related to the SNRI class of antidepressant medication. The specificity of the observed effects to SNRI pharmacological treatments requires further investigation.

**Trial registration:**

Registered at clinicaltrials.gov (NCT01051466).

**Electronic supplementary material:**

The online version of this article (doi:10.1186/s12888-015-0457-2) contains supplementary material, which is available to authorized users.

## Background

Major depressive disorder (MDD) is characterized by a prolonged low mood, neurovegetative disturbances, and cognitive impairments. Neuroimaging has aided in the delineation of the neural circuitry of MDD [1,2], determination of the effects associated with a course of therapy [3-5], provision of novel insights for neuropsychological models [2], and the potential for the development of prognostic and diagnostic biomarkers [6,7].

Within the neural circuitry of MDD, the intensity of engagement and their regional distribution depend in part on the emotional and cognitive features of the particular task. For example, in response to negative stimuli, MDD patients tend to show greater responsivity in the amygdala, dorsal anterior cingulate and insula, but reduced activity in the dorsolateral prefrontal cortex and striatum relative to healthy participants, while measures of resting state have most commonly revealed greater regional cerebral blood flow in the thalamus [5]. Studies have generally reported findings from a single task, while concurrently acquired, multiple functional and structural measures may provide a more comprehensive assessment [1-6,8]. Furthermore, longitudinal treatment studies have most frequently investigated the serotonin reuptake inhibitors (SRI), in which reduced activity in subcortical and limbic regions in MDD patients has been noted following treatment [3-5]. However, the effects of the SRI class of antidepressants may not necessarily be extrapolated to norepinephrine reuptake inhibitors (NRI) [9-12].

The present study is a multimodal investigation of the functional and structural neuroanatomy of depression in a prospective, longitudinal design with the dual serotonin-norepinephrine reuptake inhibitor (SNRI) duloxetine. MDD patients participated in MRI scans during an acute depressive episode and during the course of treatment, and healthy controls had the same scans at the same time points. Our main hypothesis was that treatment would be associated with normalization of anterior cingulate and amygdala activation in response to sad faces in MDD patients as compared with healthy participants [3-5].

## Methods

The study was approved by the Cambridgeshire 4 Research Ethics Committee, NHS Research Ethics Committee, National Research Ethics Service, NHS Health Research Authority, and all participants provided informed written consent. The study was conducted in conformity with the Declaration of Helsinki and its amendments. Study procedures and implementation were consistent with Good Clinical Practice Guidelines and all applicable regulatory requirements.

### Participants

Participants were recruited from the general community by newspaper advertisement. Inclusion criteria for all participants were an age range of 25 to 65 years and being right-handed. MDD patients met criteria for a single episode of MDD or recurrent MDD, without psychotic features, as defined by the *Diagnostic Statistical Manual of Mental Disorders*, Fourth edition, text revision (DSM-IV-TR) [13] and assessed with the Structured Clinical Interview for DSM-IV Axis I disorders (SCID-IV) [14]; were free of current antidepressant medication for a minimum of 6 weeks for fluoxetine treatment or 4 weeks for other antidepressant medication before the start of treatment at baseline (week 0); and had a 17-item Hamilton Rating Scale for Depression (HRSD-17) [15,16] total score ≥ 18 at the screening assessment and baseline. Healthy participants were matched by age, gender, and intelligence quotient (IQ); had HRSD-17 total score ≤ 7 at screening and baseline; and did not meet criteria for MDD based on SCID-IV. IQ was evaluated with the Wechsler Adult Intelligence Scale-III UK (WAIS-III UK) [17].

Exclusion criteria were any significant comorbid medical or psychiatric disorders, as defined by DSM-IV-TR Axis I or II disorder including a history of substance abuse or dependence within the prior 6 months, excluding nicotine and caffeine; known Alzheimer’s disease or mental retardation; serious suicidal risk or risk of self-harm (Columbia-Suicide Severity Rating Scale) [18]; history of electroconvulsive therapy, transcranial magnetic stimulation, or vagus nerve stimulation within the past year; abnormal thyroid stimulating hormone concentration; or medical disorders known to affect central nervous system structures or function.

Enrolled in the study were 32 MDD patients, having a moderate to severe severity of depression (mean HRSD-17 = 22.4 (standard deviation (SD) = 2.7)), and 28 healthy participants, with no significant between-group differences in demographics (Table 1). Twenty-four MDD patients and 23 healthy participants completed all the serial MRI scans.

**Table 1:** **Demographics and baseline characteristics**

	**MDD patients**	**Healthy participants**
Number	32	25^a^
Age	40.2 (11.2)	38.8 (9.9)
Age range	25.0-57.9	27.3-58.2
Male	19 (59.4 %)	12 (48.0 %)
Ethnicity		
White	18 (56.3 %)	15 (60.0 %)
Asian	10 (31.3 %)	3 (12.0 %)
African descent	4 (12.5 %)	7 (28.0 %)
Current alcohol use	22 (68.8 %)	19 (76.0 %)
Current tobacco use	6 (18.8 %)	1 (4.0 %)
HRSD-17	22.4 (2.7)	0.5 (1.3)
HAMA	21.1 (5.8)	0.4 (0.9)
WAIS-III	107.4 (11.2)	109.2 (14.6)
CGI-S	4.4 (0.6)	1.0 (0.0)
PGI-S	3.8 (1.1)	NA
SDS	19.3 (5.4)	0.2 (0.8)

All values are presented as mean and standard deviation in parenthesis, except where indicated. Age is in years. Number of participants and percentage of participants are presented for Male gender, Ethnicity, Current alcohol and tobacco use. Total scores are presented for HRSD-17, HAMA, WAIS-III and SDS. Participants were matched by age (*p* = 0.62), gender (*p* = 0.39), and WAIS-III IQ (*p* = 0.61) with no significant difference between groups, similarly for alcohol (*p* = 0.55) and drug use (*p* = 0.12). Abbreviations: CGI-S, Clinician Global Impression of Severity scale; HAMA, Hamilton Anxiety Rating Scale; HRSD-17, 17-item Hamilton Rating Scale for Depression; MDD, major depressive disorder; NA, not applicable; PGI-S, Patient Global Impression of Severity scale; SDS, Sheehan Disability Scale; WAIS-III, Wechsler Adult Intelligence Scale third UK edition.

^a^excluding 3 inadvertently enrolled healthy participants who did not meet entry criteria.

### Study design

The protocol consisted of a 12-week treatment period for MDD patients with duloxetine at a dosage of 60 mg once daily for the first 8 weeks. At week 8, MDD patients whose symptoms met criteria for remission continued taking 60 mg once daily, while those who did not had an optional dosage-increase up to 120 mg once daily (Additional file 1: Figure S1).

At baseline, MDD severity was evaluated with the following scales: SCID-IV [13], HRSD-17 [14,15], Hamilton Anxiety Rating Scale (HAMA) [19], Columbia-Suicide Severity Rating Scale (C-SSRS) [18], Clinical Global Impression of Severity scale (CGI-S) [20], Patient Global Impression of Severity scale (PGI-S) [20], and Sheehan Disability Scale (SDS) [21]. IQ was evaluated with the WAIS-III UK [17] at weeks 0, 1, or 4. At each subsequent visit, the following assessments were performed: clinical assessment and administration of HRSD-17, HAMA, CGI-S, SDS, and PGI-S by a consultant psychiatrist or senior resident in psychiatry under supervision by a consultant psychiatrist. Response to treatment was defined as a minimum of 50% reduction from the week 0 (baseline) HRSD-17 total score. Remission was defined as an endpoint HRSD-17 total score of ≤ 7. During the study, safety and tolerability to treatment was assessed through collection and monitoring of discontinuation rates, treatment-emergent adverse events, serious adverse events, vital signs, laboratory analyses, and clinical assessments including questioning of suicide-related behavior and ideations using the C-SSRS.

Healthy participants were evaluated at baseline with the following rating scales: SCID-IV, HAMA, and WAIS-III UK. All visits were reviewed with a consultant psychiatrist.

### Functional and structural MRI data acquisition

Magnetic resonance imaging (MRI) scans were acquired on a 3 Tesla GE SIGNA HDx (Milwaukee, WI, USA) at King’s College London. MRI scans were acquired at weeks 0, 1, 8, and 12 for all participants.

#### Structural MRI scan

A high-resolution 3-dimensional sagittal T1-weighted structural image was acquired at each session (Magnetization Prepared Rapid Gradient Echo; resolution 1 mm^3^). The functional MRI tasks included affective facial expressions [4,22,23], negative attentional bias task (emotional Stroop) [24], and resting state [8].

#### Affective facial expressions functional MRI task

The event-related functional MRI paradigm consisted of facial expressions and baseline trials presented in a random order [4,22,23]. Each facial stimulus was presented twice at each intensity (60 faces in total), along with 12 baseline trials consisting of a crosshair for a total of 72 presentations. Facial stimuli consisted of 10 faces (5 females) adapted from Pictures of Facial Affect by Ekman and Friesen morphed to represent varying intensities: low, medium and high [25]. Each stimulus was presented for 3 seconds. The interval between trials varied randomly according to a Poisson distribution, with a mean intertrial interval of 5 seconds, for a total duration of 360 seconds (6 minutes). Participants were instructed to specify the gender of the face (male, female), and responses were made by pressing a button.

Gradient echo T2*-weighted echoplanar images were acquired depicting blood oxygenation level-dependent (BOLD) contrast. A total of 180 volumes were acquired for each for the happy and sad facial tasks. For each volume, 39 oblique axial slices parallel to the intercommissural plane were collected with the following parameters: slice thickness: 3 mm, slice gap: 0 3 mm, echo time (TE): 30 milliseconds, repetition time (TR): 2000 milliseconds, flip angle: 75°, field of view: 240 mm, and matrix size: 64 × 64.

#### Emotional Stroop functional MRI task

The emotional Stroop task consisted of 40 negative and 40 neutral words presented in alternating blocks of eight words per emotional and neutral category, repeated five times. Each word was presented only once with a presentation time of 700 milliseconds per word. All words appeared on a dark grey background in red, blue, green, or yellow color, pseudo-randomized across the two valence categories. Four different stimulus sets which varied in the order of presentation of emotional and neutral word category blocks were randomized between scan sessions. The task was projected onto a screen and viewed from a mirror inside the scanner. Participants were instructed to name the color of the word as quickly as possible. A microphone was used to record vocal responses and to provide auditory feedback of vocal input. Reaction times to the onset of the vocal responses were measured. Verbal responses during the MRI scan were made in the absence of scanner noise as a clustered fMRI image acquisition sequence was used [24].

The emotional Stroop task was acquired in 133 T2*-weighted echoplanar images, for each volume: 39 oblique axial slices parallel to the intercommissural plane collected over 2000 milliseconds, allowing for a silent period of 2000 milliseconds in a clustered fMRI acquisition. TE: 30 milliseconds, flip angle: 90°, slice thickness: 3 mm, interslice gap: 0.3 mm, matrix size: 64 × 64. The first 4 volumes collected were acquisitions to allow for T1 equilibrium effects.

#### Resting state functional MRI

Whole-brain functional resting state data were collected while participants were instructed to stay awake with their eyes closed and not to think of anything specific. Scan duration was 8.5 minutes. T2*-weighted single-shot gradient echo echoplanar sequence was acquired with the following parameters: TE: 30 milliseconds, TR: 2 seconds, FA: 75°, voxel size, 3.75 × 3.75 × 3.3 mm. Headphones and cushions were used to minimize scanner noise and head motion, respectively.

### Pre-specified primary outcome measure and secondary analyses

The pre-specified primary outcome measure was the mean percentage signal change in functional MRI BOLD contrast response from baseline to week 12 in the mean of the right and left amygdalae, in response to sad facial affect processing, comparing MDD and healthy participants. The sample size for the study was based on effect size estimates for this primary outcome, obtained from our previous work on pre- to post- SRI treatment effects on amygdala activation in MDD patients relative to healthy controls [4].

Secondary outcomes included baseline-to-endpoint changes in illness severity, as assessed by HRSD-17, HAMA, CGI-S, Patient Global Impression of Improvement scale, and SDS global functioning impairment score, and their correlation with changes in structural and functional correlates over sessions in the following regions of interest: anterior cingulate cortices, amygdalae, and hippocampi. Changes in functional MRI BOLD contrast response and volumes of each region of interest from week 0 to weeks 1, 8, and 12 were analyzed using a restricted maximum likelihood-based mixed-effects model repeated measures (MMRM) approach. The model included the categorical effects of group, visit, and group-by-visit interaction as well as the continuous covariate of baseline measurement. Significance tests were based on least-square mean changes and Type III sum-of-square, implemented using SAS PROC MIXED (SAS, version 9 1, Cary, NC, USA). Logistic regression was also used to examine the association between endpoint remission and changes in neural correlates. The region-of-interest analyses were performed in all enrolled participants, using MMRM model or last observation carried forward (LOCF) methodology for missing observations (eg. participants who did not complete the study). No multiple comparisons correction procedures were applied to the MMRM analyses as these were pre-specified.

As well, functional whole-brain image analyses were conducted on a complete case basis involving each scan session (ie. with participants who participated in all four MRI scans) as standard software for whole-brain neuroimaging analysis does not permit “missingness” in the data set of images. As explained in detail below, whole-brain image analyses were focused on functional changes over time in the treatment and control samples, as well as prediction of treatment improvement (with HRSD-17 or HAMA) from baseline functional measurements. Complete data available for each task were varied due to scan acquisition difficulties, such as excessive movement during the scan and late arrival of participants leading to incomplete scan sessions. The number of participants who completed these tasks for all the scan sessions: happy and sad faces (23 MDD and 23 healthy participants); emotional Stroop (21 MDD and 20 healthy participants); and resting state (21 MDD and 20 healthy participants). Behavioral data are presented in the Additional file 1.

### Functional and structural MRI analysis

#### Structural MRI analysis

Analysis of the structural images was performed with Freesurfer 4.5.0 automated longitudinal stream to obtain the volumes of *a priori* regions of interest: anterior cingulate cortices, amygdalae, and hippocampi [26]. Quality control was performed by visually assessing each Freesurfer brain segmentation overlaid on the original T1 image to ensure that cortical reconstructions did not present major anomalies. The medial temporal lobe region was assessed with coronal sections. All reconstructions passed this qualitative control, and the original Freesurfer outputs were used without manual corrections. High intraclass correlations (ICC) for repeated measurements were observed for all the volumetric measurements in the healthy control participants (all > 0.91) (Additional file 1: Table S1). Volumetric measurements of the amygdalae, hippocampi and anterior cingulate were included in second-level MMRM and logistic regression models.

#### Functional MRI analysis: task-related data

Statistical Parametric Mapping (SPM8, Wellcome Department of Imaging Neuroscience, London, UK) was used to preprocess and analyze the task-related fMRI data. Images were realigned to correct for motion artifacts, spatially normalized to the Montreal Neurological Institute template, and smoothed using an 8-mm full-width at half maximum Gaussian kernel filter. Group analysis used a random effects model consisting of a 2-stage hierarchical procedure with the first-level analysis performed by using the general linear model, accounting for serial autocorrelations by application of an autoregressive model.

#### Affective facial expressions task

In the sad and happy faces tasks, stimuli presentations were modeled as individual events, and the first-level analysis produced contrast images relevant to the main contrast of interest (sad faces or happy faces vs. crosshair baseline). For the primary outcome measure, the MarsBar SPM toolbox was used to estimate mean activation in the *a priori* regions of interest.

#### Emotional Stroop task

In the emotional Stroop task, the first-level analysis produced individual mean images corresponding to the main contrast of interest (negative > neutral) and the time series was modeled as a block-design.

#### Second-level analysis of task-related functional tasks

For each task, its second-level analysis employed a random-effects model to examine the main effect of group (MDD vs. healthy participants across all time points), main effect of time (linear changes over weeks 0, 1, 8, and 12) and the group by time interaction. T-tests were also used to compare scanning data at a particular time point between groups. Inference of whole-brain statistical images was conducted using the general linear model and cluster-wise family-wise error rate control with *p*< 0.05 corrected for multiple comparisons. For *post hoc* analyses only, in order to identify the direction of changes responsible for an interaction effect, less conservative thresholds were also employed as indicated in the Results section.

### Functional MRI analysis: resting-state data

Resting-state analysis was performed using FMRIB Software Library (FSL) v5.0 (http://fsl.fmrib.ox.ac.uk/fsl/fslwiki/). Preprocessing included motion correction, skull stripping, spatial smoothing at 5 mm full-width at half maximum, and registration to standard space. Extraction of resting-state networks at the group level was conducted by using FSL Multivariate Exploratory Linear Optimized Decomposition into Independent Components (MELODIC) [27]. MELODIC was set to estimate 25 components to extract stable connectivity estimates of the default mode networks (DMNs) [8]. Five independent components depicting DMN activity were identified (Additional file 1: Figure S2) [28], encompassing the canonical default mode inclusive of the two core regions (anterior medial prefrontal and posterior cingulate cortices), dorsomedial prefrontal subsystem (dorsomedial prefrontal cortex, lateral temporal cortex, and temporoparietal junction), and medial temporal lobe subsystem (ventromedial prefrontal cortex including ventral cingulate, parietal lobule, retrosplenial cortex, and hippocampal formation) [29,30]. Dual regression was used to generate participant-specific and scan session–specific versions of group-level DMN spatial maps in two stages, resulting in a set of participant-specific spatial maps for each scan session and participant. *Second-level analysis of resting-state data:* Scan-specific maps were used to estimate contrast maps depicting linear changes across successive scans for each participant. These statistical maps (one per participant) were entered in a higher-level general linear model analysis, and statistical inference was performed with nonparametric permutation testing [31]. Correction for multiple comparisons was conducted using threshold-free cluster enhancement with family-wise error (FWE) rate control with p < 0.05 corrected for multiple comparisons [32].

## Results

### Clinical measures

MDD patients showed a significant improvement in their depression, as assessed by changes in HRSD-17 (−13.9 [7.0]); HAMA (−11.5 [8.6]); SDS global functioning impairment score (−9.8 [8.9]); and CGI-S (−2.2 [1.3]). Upon study completion at week 12, 18 MDD patients (75.0% of MDD completers) fulfilled criteria for remission and 19 MDD patients (79.1%) fulfilled criteria for clinical response. Applying the last observation carried forward analysis with inclusion of all enrolled participants, there were no significant differences in the history of depression between responders (n = 20, median 1 episode, mean 2.7 [4.43]) and non-responders which included MDD participants who did not complete the study (n = 7, median 2 episodes, mean 6.14 [10.53]) (p = 0.43). The frequency and nature of adverse events were consistent with the known profile of duloxetine [33], and there was one serious adverse event of retinal pigment epitheliopathy which was not judged to be related to the study or duloxetine.

### Structural magnetic resonance imaging

There were no significant group by time effects nor any baseline differences in anterior cingulate cortices, amygdalae, or hippocampi volumes (Additional file 1: Table S1).

### Affective facial expressions

Contrary to our hypothesis, there were no significant between group differences in the change in BOLD response from baseline to sad faces as analyzed with the MMRM approach nor any significant group by time effects from the whole-brain analysis. There were no significant differences between groups at baseline (Additional file 1: Table S1).

Within the MDD group, a main effect of time was observed in which there was a significant increase in the BOLD response to the mean of the medium and high intensity of expressions in the posterior cingulate/precuneus (x = −3, y = −43, z = 19; 221 voxels; peak T = 4.50; p (FWE corrected) = 0.010), while healthy participants showed a trend towards a decrease in the orbitofrontal region (x = 45,y = 29, z = 11; 118 voxels, T = 4.61, p (FWE corrected) = 0.068).

Similarly, no significant group by time effects or any baseline differences between groups were observed in the happy faces task. There were no main effects of time in the MDD patients, but healthy participants showed a significant decrease with time in response to the mean of medium and high intensity of expressions in the anterior cingulate (x = 9, y = 29, z = 40; 315 voxels, peak T = 4.27; p (FWE corrected) = 0.002) and precentral region (x = −51, y = 11, z = 34; 190 voxels; T = 4.08; p (FWE corrected) = 0.018), as well as approaching significance in the thalamus (x = 3, y = −13, z = 10; 118 voxels; T = 4.12; p (FWE corrected) = 0.070).

### Emotional Stroop

**Figure 1:**
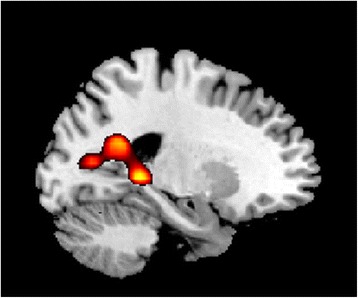
Emotional Stroop. A significant group by time effect was found for the emotional Stroop in the posterior cingulate extending into the precuneus.

A significant group by time interaction was observed in the left posterior temporoparietal junction involving the parahippocampal cortex (x = −18, y = −40, z = 1; 414 voxels; peak T = 4.11; p (FWE corrected) = 0.014) as well as precuneus and posterior cingulate cortex (subordinate peaks at x = −24, y = −52, z = 22 and x = −21, y = −70, z = −10) during the processing of negative relative to neutral words (Figure 1). The interaction effect was found to be driven by reductions observed in MDD patients (significant at p = 0.001 uncorrected) with successive scans relative to healthy participants who showed no significant changes with time. At baseline, there was a main effect of group in which MDD patients showed greater activation relative to healthy participants in a region including the posterior cingulate cortex and precuneus bilaterally (right: x = 9, y = −43, z = 19; left: x = −15,y = −43, z = 4, and x = 15, y = −49, z = 13; -134 voxels; peak T = 4.51; p (FWE corrected) = 0.026).

### Resting state

**Figure 2:**
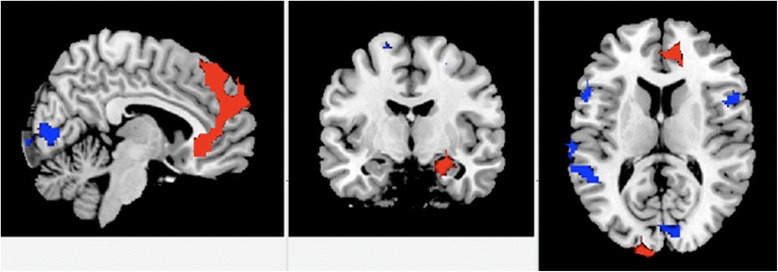
Resting-state functional magnetic resonance imaging. Linear changes in resting-state functional fMRI with successive scans, Areas with reductions in connectivity to the default mode network (DMN) regions with time are shown in blue, and areas with increased connectivity to the DMNs are depicted in red.

No significant group by time effects were found, but main effects of time were observed within each group. MDD patients showed decreased connectivity with successive scans (Figure 2) between DMN components and bilateral prefrontal cortices, namely with right dorsolateral (IC06; x = 52, y = 10, z = 18; 118 voxels; T = 3.9; 117 voxels; p (FWE corrected) = 0.034), right superior frontal premotor cortex (IC06; x = 22, y = −2, z = 64; T = 4.25; 41 voxels; p (FWE corrected) = 0.030), and left inferior frontal gyrus (IC06; x = −54, y = 14, z = 16; T = 4.79; 36 voxels; p (FWE corrected) = 0.018), as well as decreased connectivity between DMN components and auditory processing cortex (IC10; x = −57, y = −48, z = 19; T = 5.85; 1078 voxels; p (FWE corrected) = 0.007), and primary visual and extrastriate regions (IC20; x = 2, y = −78, z = 4; T = 4.88; 492 voxels; p (FWE corrected) = 0.005). Increases in connectivity between components of the DMN in MDD patients were found with medial prefrontal regions, including pregenual and subgenual cingulate and the frontal pole (IC08; x = 10, y = 30, z = −8; T = 5.04; 7287 voxels; p (FWE corrected) = 0.007), right hippocampus (IC24; x = 42, y = 14, z = −36; T = 4.13; 30 voxels; p (FWE corrected) = 0.023), parahippocampal gyrus (IC24; x = 42, y = −30, z = −20; T = 4.05; 431 voxels; p (FWE corrected) = 0.035), angular gyrus (IC08; x = 54, y = −46, z = 24; T = 4.99; 190 voxels; p (FWE corrected) = 0.010), and middle occipital gyrus (IC08; x = 10, y = −102, z = 8; T = 5.69; 263 voxels; p (FWE corrected) = 0.009). Healthy participants showed decreased connectivity with time between the DMN with the posterior hippocampus extending into the fusiform region (IC06; x = 30, y = −38, z = 0; T = 4.83; 45 voxels; p (FWE corrected) = 0.027). There was also increased connectivity with time in healthy participants between the DMN and posterior cingulate (IC08; x = 6, y = −50, z = 8; T = 3 78; 85 voxels; p (FWE corrected) = 0.030), fusiform gyrus (IC08; x = 34; y = −38, z = −12; T = 4.61; 375 voxels; p (FWE corrected) = 0.010), superior medial frontal gyrus (IC08; x = 2; y = 34, z = 36; T = 3.85; 91 voxels; p (FWE corrected)- = 0.029), premotor cortex (IC08; x = −26; y = 10, z = 52; T = 4.19; 91 voxels; p (FWE corrected) = 0.025), and parietal lobule (IC08; x = 50; y = −54, z = 44; T = 4.30; 808 voxels; p (FWE corrected) = 0.006).

### Predictors of clinical response

**Figure 3:**
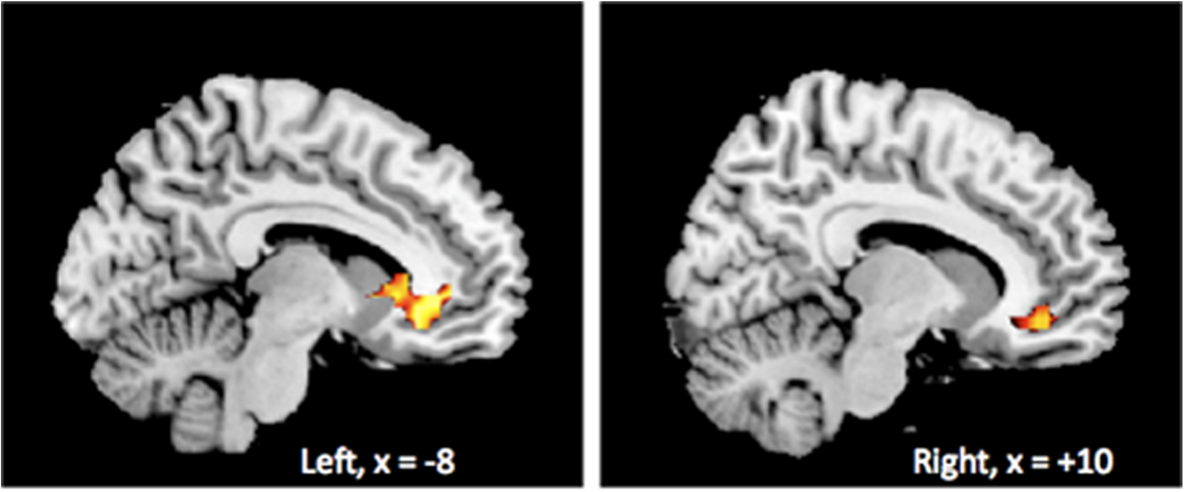
Baseline connectivity in ventral cingulate and orbitofrontal resting state network. Decreased baseline connectivity in ventral cingulate and orbitofrontal resting-state network predicted an improved response in correlation with the normalized change in HRSD-17 score from week 0 to week 12 corrected for multiple comparisons.

**Figure 4:**
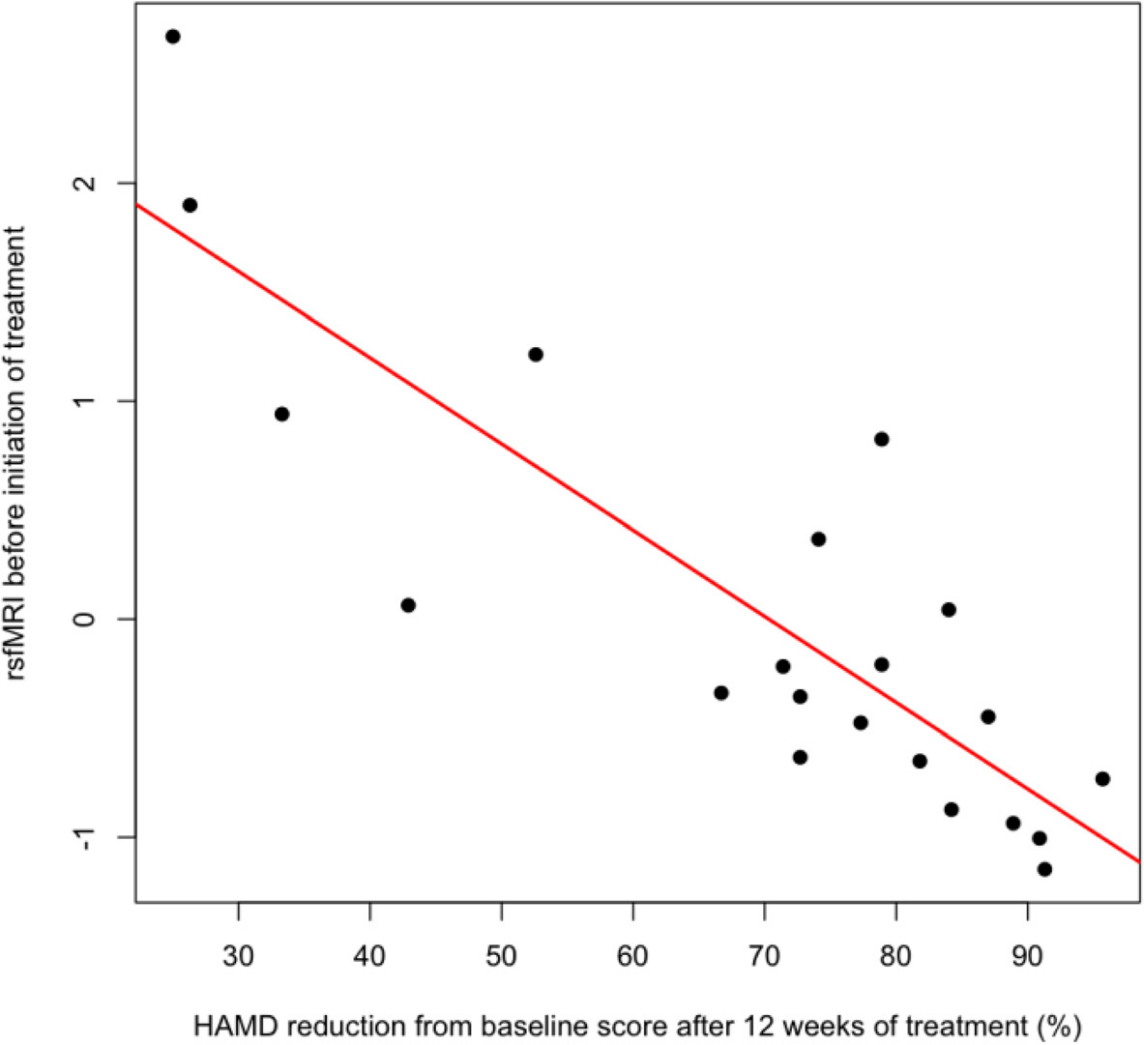
Association between baseline connectivity and change in depressive severity. Scatter plot of baseline resting-state fMRI baseline connectivity activity in subgenual cingulate and clinical response to 12 weeks of treatment with duloxetine as measured by the normalized change in HRSD-17 score from week 0 to week 12.

Baseline resting-state activity within the orbitofrontal component of the DMN in MDD patients, before treatment was initiated, was negatively correlated with improvement with treatment as measured by HRSD (Figures 3 and 4). MDD patients with reduced connectivity in the orbitofrontal component of the DMN (BA10/25/47) (left subgenual anterior cingulate (BA 25/11): x = 6, y = 30, z = −10; T = 6.84, 691 voxels; p (FWE corrected)- = 0.003; right subgenual/pregenual anterior cingulate: x = 12, y = 42, z = 8; T = 5.56; 83 voxels; p (FWE corrected)- = 0.021) showed the greatest improvement with treatment. No other functional MRI or structural baseline measures were correlated with changes in HRSD or HAMA based on the whole-brain analysis.

From the MMRM model, which accounted for participants who had not completed all the scans with a last observation carried forward methodology, an early increase in left hippocampal volume after 1 week of treatment predicted clinical remission following 12 weeks of treatment (odds ratio 1.01 (95% CI: 1.00, 1.02) p = 0.031) (Additional file 1: Table S2- S3). High intraclass correlations for repeated measurements were observed for all the volumetric measurements in the healthy control participants (all > 0.91), which were 0.976 and 0.961 for the right and left hippocampi, respectively (Additional file 1: Table S1).

## Discussion

Distinct neural effects of treatment with duloxetine were revealed in resting state connectivity, affective facial processing, and negative attentional processing. Contrary to our hypothesis, we did not find any group by time interaction effects in the neural responses to sad facial expressions [3-5]. Instead, marked effects in the posterior cingulate cortex were evident in response to a task designed to engage the negative attentional bias in MDD [24], and there were time-dependent changes in the DMN in MDD patients in which increased connectivity towards limbic regions but decreased connectivity with lateral cortical regions emerged as treatment progressed. Furthermore, baseline resting state connectivity within the orbitofrontal component of the DMN, namely in the bilateral anterior cingulate regions, was a significant a predictor of clinical response.

Normalization of limbic hyper-responsiveness has been commonly reported in MDD [3-5] and appears to be specific to sad facial expressions [34]. However, we did not observe increased amygdala activation to sad faces in the acutely depressed MDD participants nor any significant group by time effects following treatment. Potential confounds include factors related to the sample and task. In the present group, the depressive symptoms was of a moderate to severe severity which is comparable to previous samples in which increased amygdala responses have been observed [3-5,34]. The present task used implicit affective processing in order to increase the potential to engage amygdala responsivity, while a masked presentation may have more fully captured amygdalar automatic processing [35,36], and the number of subjects and the design of the task, which was an event-related design rather than a blocked design, may have limited the power to observe a significant effect [35]. Furthermore, most studies to date have examined the effects of the SRI class of antidepressants [3-5,34]. Single doses of SRI medications in healthy participants have been associated with decreased amygdala responses to emotional faces, while single-dose NRIs lead to increased activation in medial and frontal regions [11]. It is unclear whether the effects of different classes of antidepressants are comparable as it has been proposed that SRIs have an early attenuating effects on emotional reactivity while NRIs have a more modulatory effect on attention regulation of emotional processes and may not necessarily have a direct impact on amygdala responsivity which would be observed in addition to potential state effects related to acute depressive states as compared to states of remission [9-11,37].

In order to examine the negative attention bias in MDD [38], we applied an emotional Stroop task [24,39]. We found a significant interaction effect in the posterior cingulate cortex in which increased baseline activation in MDD showed a linear normalization with successive measures following treatment as compared to healthy participants who underwent the same scans. The posterior cingulate cortex is involved in the DMN, which has a central role in many situations whereby attention is internally directed such as in episodic memory retrieval and inner reflection [40]. Increased posterior cingulate activation in MDD patients while acutely depressed may be understood as reflecting a failure to attenuate self-referential activity, perhaps leading to interference in task performance. With treatment, attenuation of posterior cingulate activity may reflect an improvement in selective attention and the ability to focus.

In parallel, the resting-state functional connectivity in MDD patients showed increased connectivity over the course of treatment within the anterior DMN in the subgenual anterior cingulate and regions involved in attention-processing, namely the superior frontal and parietal cortices, while reduced connectivity was observed in the prefrontal regions linked to the DMN. Anand *et al.* [41] also found increased connectivity with the anterior cingulate and limbic regions following treatment with a variety of antidepressant medications, and Li *et al.* [42] have proposed that persistent increased functional connectivity in anterior DMN reflects a trait effect of MDD and a potential risk for relapse.

The present findings bring into question the potential for amygdala responsivity as a state marker of MDD because no significant differences were found during an acute episode or following 12 weeks of treatment in which the majority of patients’ symptoms fulfilled criteria for clinical remission reflecting the numerous factors which impact on amygdala responsivity [35]. Rather the negative affective bias appears to have been more strongly detected by the emotional attention processing task which revealed a significant group by time effect with normalization of activation in the posterior cingulate. The corresponding increase in resting state connectivity in MDD patients with treatment highlights potential links between the negative affective bias that is characteristic of MDD and the resting state network [37]. Moreover, there are persuasive indications that these effects may be related to the NRI class of antidepressant medication [9-12,37] although this requires further investigation.

As a potential marker of clinical response, we found that MDD patients with reduced functional connectivity with the subgenual anterior cingulate showed the greatest clinical improvement following treatment. The subgenual anterior cingulate has a key role in MDD [43], and activity in this region has been consistently implicated as a predictor of clinical response [7,44]. Increased functional connectivity with the subgenual anterior cingulate has been associated with increased length of illness [45], and the neuropsychological mechanisms of rumination and brooding have been correlated with increased connectivity between the subgenual anterior cingulate and posterior cingulate [46], including in treatment-naïve MDD patients with increased functional connectivity in the medial prefrontal and subgenual anterior cingulate [47]. Anterior cingulate-limbic white matter tracts have also been predictive of clinical response [48], though the degree to which white matter tract structural connectivity form the basis of resting state functional connectivity requires further validation [49].

From the MMRM model, an early increase in left hippocampal volume after 1 week of treatment predicted subsequent clinical response. Although the volume change was small, the high intraclass correlations in hippocampal volumes with the repeated measures in the healthy participants indicate a high reliability of the measure. Sämann [50] reported that increased left hippocampal gray matter volume was predictive of treatment response to a variety of antidepressant medications, and our meta-analysis supported the observation of reduced right hippocampal volume being predictive of a poorer clinical response [7]. Increases in hippocampal volume have been observed following short term [51] and long term [52] treatments with antidepressant medications. Our finding suggests that antidepressant medications can increase hippocampal volume early in the course of treatment, such increases may be predictive of clinical response, and provides some corroboration for hippocampal neurogenesis as a mechanism for the effects of antidepressant therapy [53].

### Limitations

The high response rate in this open study though has limited the power to detect differences between responders and MDD patients with a more treatment-resistant form of depression, which may be associated with distinct neural correlates [41]. The absence of a placebo-control treatment arm limits our attribution of effects to the antidepressant medication as opposed to changes associated with clinical improvement, although possible confounds of time were accounted for by healthy participants having the same serial scans. Furthermore, we did not find any significant differences between MDD patients and healthy participants in response to the happy and sad faces stimuli, perhaps in part reflecting the poor test-retest reliability of amygdala response to these emotional faces [54], while resting-state fMRI data show greater robustness and reproducibility [55]. Test-retest reliability of a neuroimaging measure becomes particularly important in the development of biomarkers for prognosis and diagnosis [44].

## Conclusions

In summary, multimodal functional and structural neuroimaging correlates demonstrated significant effects of treatment in the anterior DMN associated with resting state connectivity and in response to negative attentional biases, but not in response to happy or sad facial expressions. Moreover, anterior cingulate functional connectivity predicted clinical response. Our findings reflect the distinct effects of the SNRI class of antidepressants as well as methodological factors of test-retest reliability and reproducibility of fMRI tasks. Further investigation is required to examine the specificity of the SNRI effects.

## Availability of supporting data

The data sets supporting the results of this article are included within the article and its additional files.

## Additional file

Additional file 1:
**Supplementary Methods and Results.**

## Abbreviations

BOLD: Blood oxygenation level-dependent
CGI-S: Clinical Global Impression of Severity
DMN: Default mode network
DSM-IV-TR: *Diagnostic Statistical Manual of Mental Disorders*, Fourth edition, text revision
FWE: Family-wise error
fMRI: Functional magnetic resonance imaging
FSL: FMRIB Software Library
HAMA: Hamilton Anxiety Rating Scale
HRSD-17: 17-item Hamilton Depression Rating Scale
IQ: Intelligence quotient
MDD: Major depressive disorder
MELODIC: Multivariate Exploratory Linear Optimized Decomposition into Independent Components
MMRM: Mixed-effects model repeated measures
MRI: magnetic resonance imaging
NRIs: Norepinephrine reuptake inhibitors
SCID-IV: Structured Clinical Interview for DSM-IV Axis I disorders
SDS: Sheehan Disability Scale
sMRI: Structural magnetic resonance imaging
SNRI: Serotonin-norepinephrine reuptake inhibitor
SRIs: Serotonin reuptake inhibitors
