# Supplementary material for: Multimodal functional and structural neuroimaging investigation of major depressive disorder following treatment with duloxetine
Source: BMC Psychiatry. 2015 Apr 14;15:82. doi: 10.1186/s12888-015-0457-2 (PMC4417267; doi:10.1186/s12888-015-0457-2)

### SUPPLEMENTARY MATERIALS

*Prospective multimodal functional and structural neuroimaging investigation of major depressive disorder following treatment with duloxetine. CHY Fu, SG Costafreda, A Sankar, TM Adams, MM Rasenick, P Liu, R Donati, LA Maglanoc, P Horton, LB Marangell.*

### Supplementary Results

### Behavioral Responses

### Sad faces task

In response latency, there was a main effect of intensity in which both groups showed a slower response to increasing intensity of expression (F (2,86)=14.96, p<0.001), while there were no significant main effects of time or group, nor any significant second or third order effects.

In accuracy, there were main effects of time in which more errors were made with the successive scans over time (F (3,129)=3.54, p=0.017) and of intensity in which the most errors were made for the highest intensity of expression (F (2,86)=3.744, p=0.028). There were no main effects of group, nor any significant second or third order effects.

### Happy faces task

In response latency, there was a main effect of time (F (3,123)=3.30, p=0.023) as the response latencies decreased with successive scans over time. There were no significant effects of intensity or group, nor any significant second or third order effects.

In accuracy, there were no significant main effects of time, intensity, or group. There was a significant interaction effect of intensity by group (F (2,82)=3.85, p=0.025), as MDD participants showed the greatest errors for the medium intensity of expression while healthy participants had greater errors for the lowest intensity, as well as a significant interaction effect of intensity by time (F (6,246)=2.86, p=0.01) in which the most errors were made for the medium and highest intensities at the final (week 12) scan while there were few changes in accuracy for the lowest intensity with successive scans over time.

### Emotional Stroop task

In response latency, there was a main effect of valence (F (1,40)=45.23, p<0.001), in which both groups were slower to respond to the negatively valenced words, and a main effect of group (F (1,40)=8.25, p=0.006) as major depressive disorder (MDD) participants were slower than healthy participants, but there was no main effect of time. Significant 2-way interaction effects were found for valence by group (F (1,39)=5.69, p=0.02), as MDD participants showed even greater response latencies for negative words relative to healthy participants, and valence by time (F (3,120)=4.01, p=0.009) in which there was increasing response latency for the neutral words with successive scans over time while the response latency for the negative words was greater at all time points with the slowest response at the baseline (week 0) scan followed by a faster response and subsequent slowing with successive scans.

In accuracy, there was a main effect of valence (F (1,39)=7.37, p=0.010) as both groups showed greater accuracy for neutral words, while there was no main effect of group and a trend towards a main effect of time (F (3,120)=2.46, p=0.066) with improved accuracy with time. There was also a significant valence by group interaction (F (1,39)=7.69, p=0.008), in which healthy participants showed no differences in errors between neutral and negatively valenced words while MDD participants showed greater errors for the negative words. There were no other significant second or third order effects.

### Supplementary Table 1. Structural and Functional MRI responses at baseline and Intraclass Correlations for structural MRI regions in healthy participants

|  | **MDD Patients**  **N=32** | **Healthy Participants**  **N=25** | p-**value** |
| --- | --- | --- | --- |
| **sMRI volume** |  |  |  |
| Anterior cingulate | 9333.5 (1348.2) | 9350.3 (1584.7) | 0.967 |
| Left amygdala | 1535.6 (160.7) | 1502.1 (169.1) | 0.468 |
| Right amygdala | 1698.8 (223.6) | 1732.2 (236.3) | 0.604 |
| Left hippocampus | 4314.3 (426.5) | 4179.2 (336.4) | 0.216 |
| Right hippocampus | 4310.6 (398.4) | 4210.9 (394.4) | 0.370 |
|  |  |  |  |
| **fMRI BOLD contrast responseto sad facial expressions** | | | |
| Anterior cingulate | -0.07 (0.49) | -0.15 (0.51) | 0.565 |
| Left amygdala | 0.42 (0.60) | 0.62 (0.67) | 0.239 |
| Right amygdala | 0.19 (0.52) | 0.42 (0.58) | 0.129 |
| Left hippocampus | 0.23 (0.37) | 0.35 (0.47) | 0.324 |
| Right hippocampus | 0.19 (0.41) | 0.29 (0.46) | 0.392 |
|  |  |  |  |
| **Intraclass Correlations** |  |  |  |
| Anterior cingulate | n/a | 0.954 |  |
| Left amygdala | n/a | 0.911 |  |
| Right amygdala | n/a | 0.936 |  |
| Left hippocampus | n/a | 0.961 |  |
| Right hippocampus | n/a | 0.976 |  |
|  |  |  |  |

Mean values and standard deviations in parentheses are presented. Units of measure for sMRI volumes are cm^3^ and for fMRI BOLD response are in %. Abbreviations: BOLD, blood oxygenation level-dependent; fMRI, functional magnetic resonance imaging; MDD, major depressive disorder; N, number of participants; n/a, not applicable; sMRI, structural magnetic resonance imaging.

### Supplementary Table 2. Pearson correlation between change in neuroimaging and clinical measures (last-observation-carried-forward analysis) in MDD patients (N=28)

| **Region** | **Study Interval** | **Endpoint Measure** | ***r*** | **p-value** |
| --- | --- | --- | --- | --- |
| fMRI BOLD anterior cingulate | Baseline | HAMA total score | -0.403 | 0.034 |
|  | Baseline to week 8 |  | 0.427 | 0.024 |
|  | Baseline to week 12 |  | 0.456 | 0.015 |
| fMRI BOLD anterior cingulate | Baseline to week 8 | CGI-S | 0.405 | 0.032 |
|  | Baseline to week 12 |  | 0.359 | 0.61 |
| fMRI BOLD anterior cingulate | Baseline | PGI-I | -0.458 | 0.014 |
|  | Baseline to week 1 |  | 0.450 | 0.016 |
|  | Baseline to week 8 |  | 0.343 | 0.074 |
|  | Baseline to week 12 |  | 0.436 | 0.020 |
| sMRI volume left hippocampus | Baseline to week 1 | HRSD-17 total score | -0.339 | 0.077 |
|  | Baseline to week 12 |  | -0.300 | 0.120 |

Abbreviations: BOLD, blood oxygenation level-dependent [contrast]; CGI-S, Clinical Global Impression of Severity scale; fMRI, functional magnetic resonance imaging; HRSD-17, 17-item Hamilton Rating Scale for Depression; LOCF, last observation carried forward; MDD, major depressive disorder; N, number of participants; PGI-I, Patient Global Impression of Improvement scale; sMRI, structural magnetic resonance imaging

### Supplementary Table 3. Prognosis classifier and week 12 clinical remissionin MDD patients (N=28) (last-observation-carried-forward analysis)

| **Neuroimaging Measure** | **Study Interval** | **Odds Ratio^a^ (95% CI)** | **p-value** |
| --- | --- | --- | --- |
|  |  |  |  |
| sMRI volume left hippocampus | Baseline to week 1 | 1.01 (1.00, 1.02) | 0.031 |
|  | Baseline to week 12 | 1.01 (1.00, 1.02) | 0.042 |

^a^Odds ratio corresponding to a 10 mm^3^ increase in volume

Abbreviations: CI, confidence interval; LOCF, last observation carried forward; MDD, major depressive disorder; N, number of participants; sMRI, structural magnetic resonance imaging

### Supplementary Figure 1. Study Design


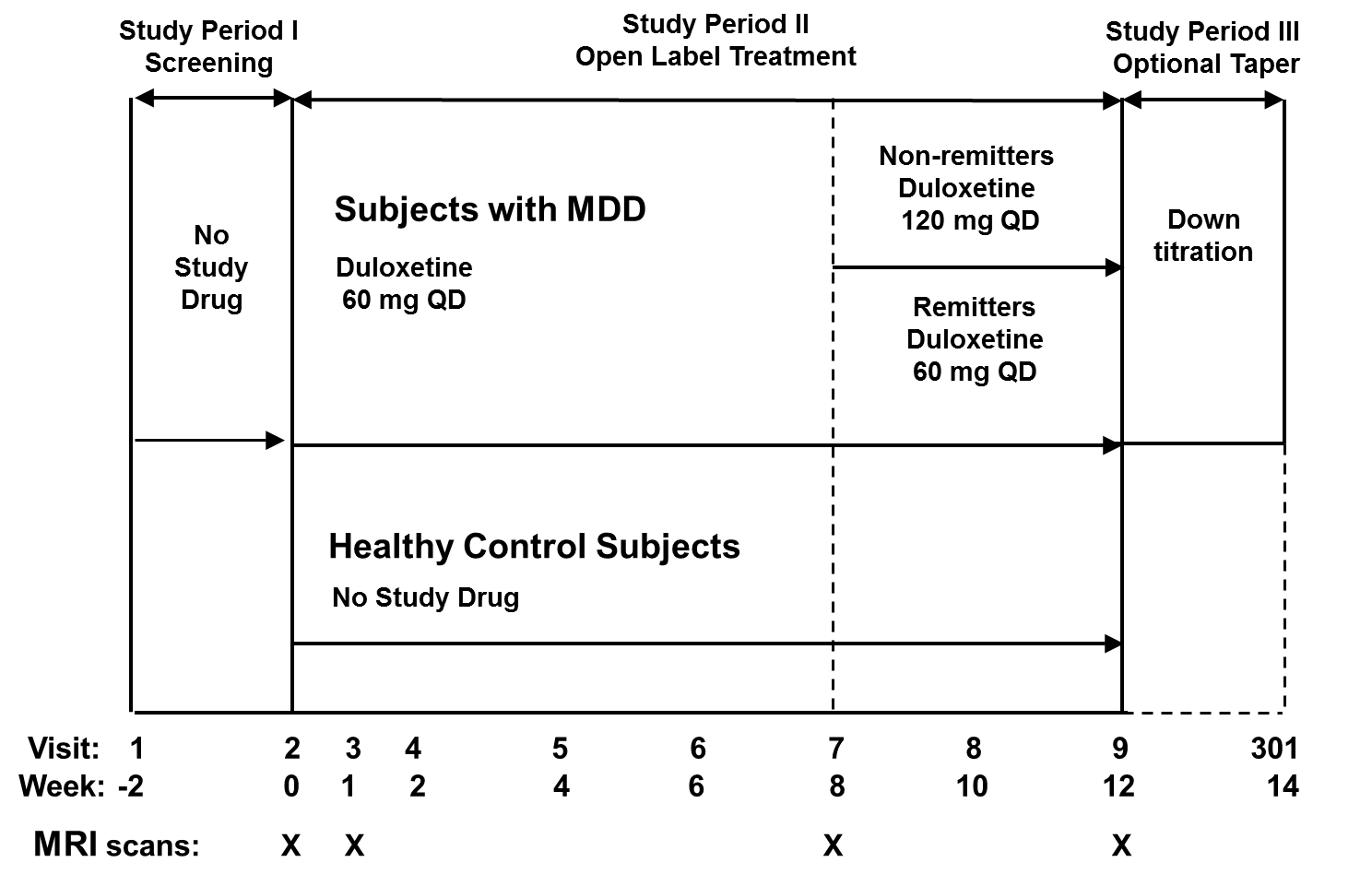


Abbreviations: MDD, major depressive disorder; MRI, magnetic resonance imaging; QD, once daily

### Supplementary Figure 2. Independent component analysis of resting-state fMRI revealed five component networks representing core areas of the default mode network.


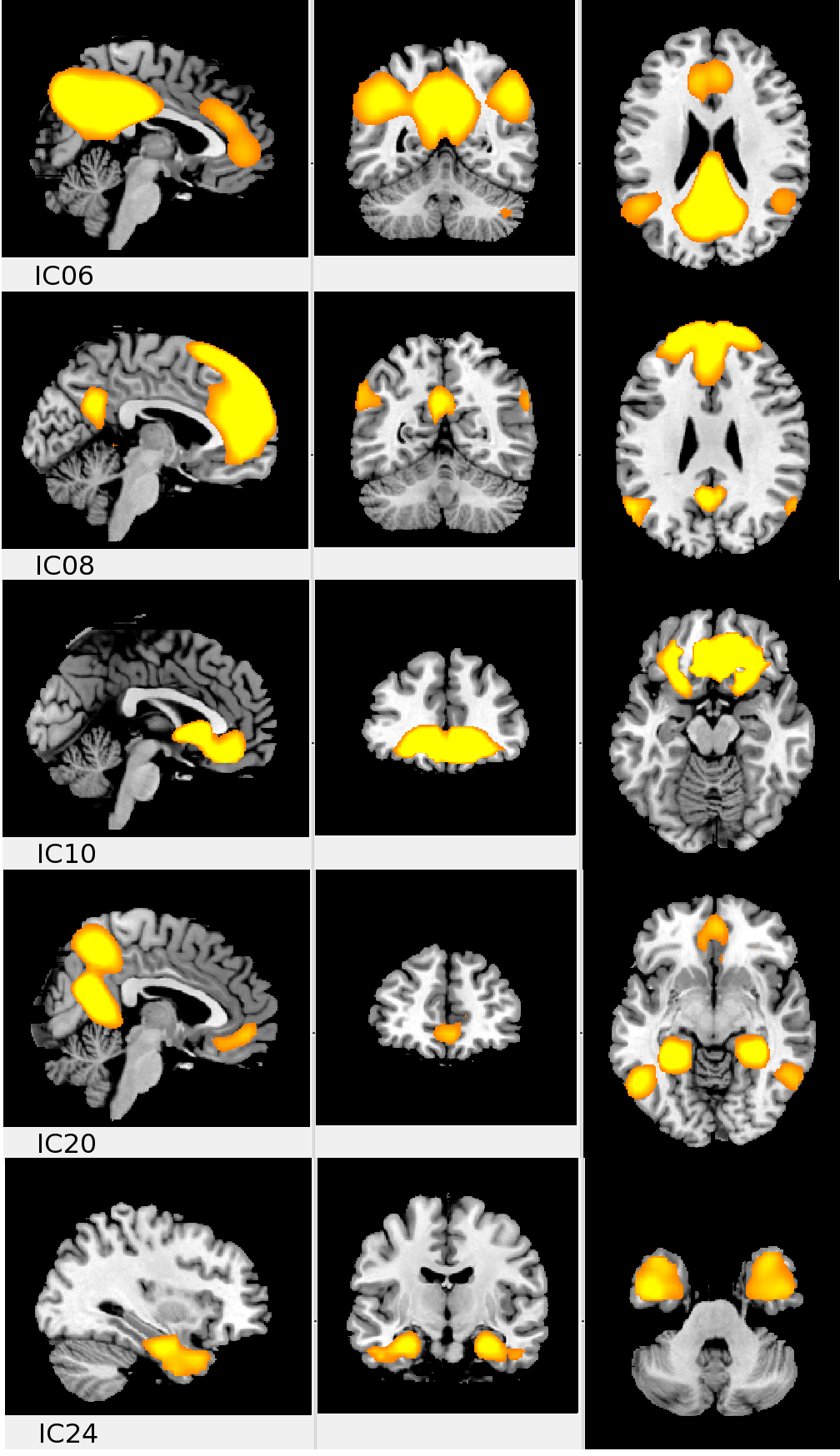

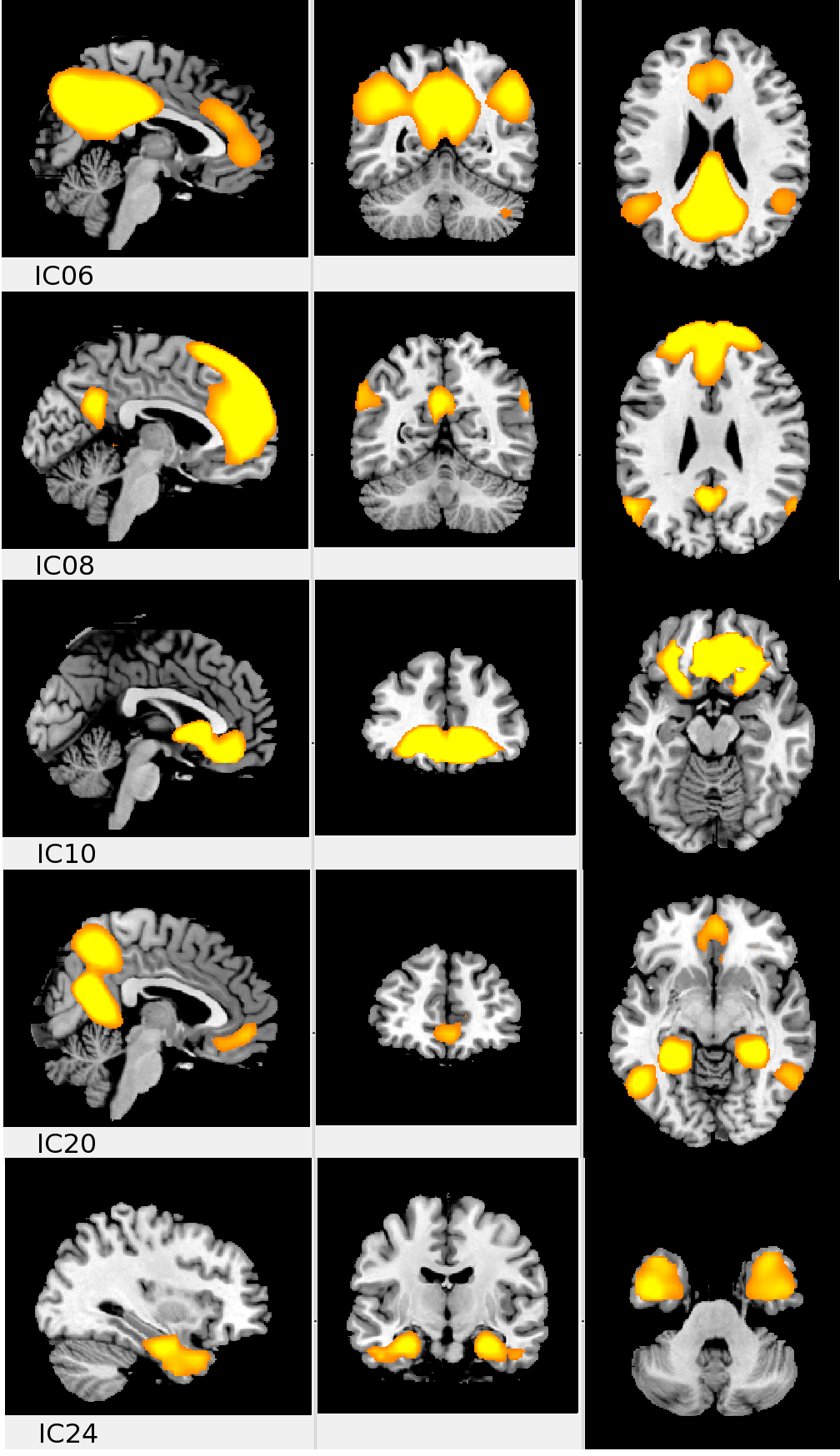

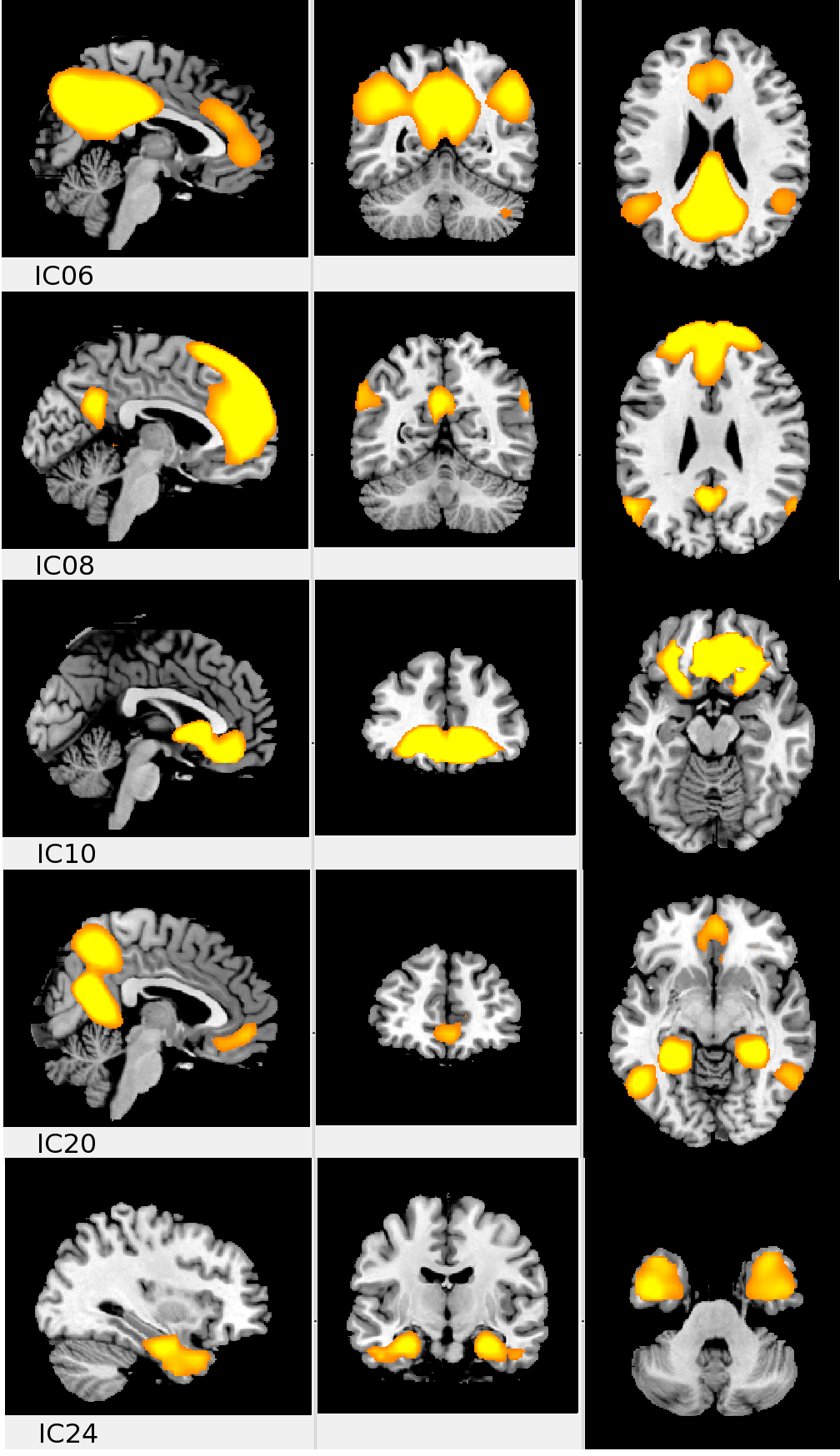

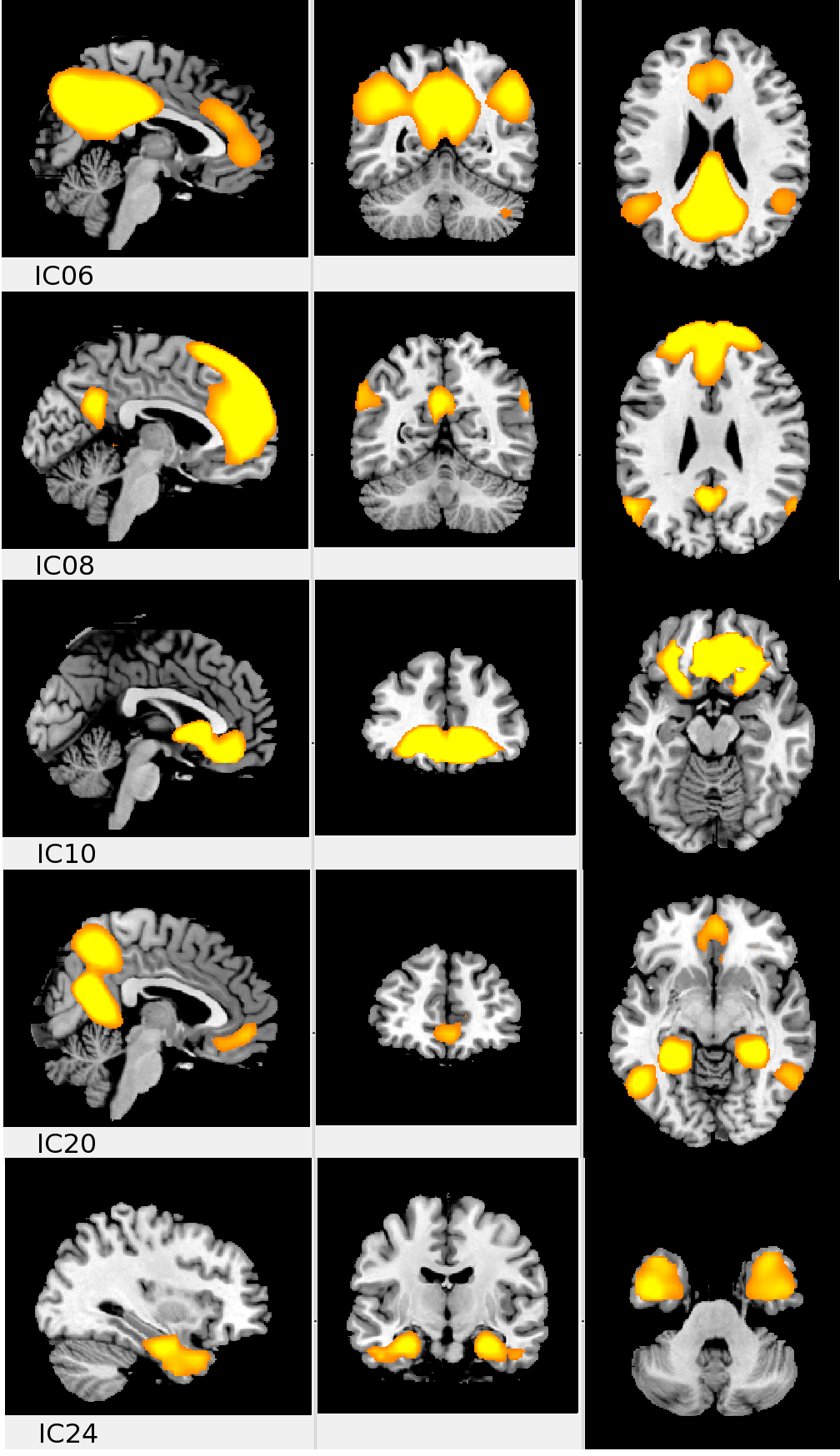

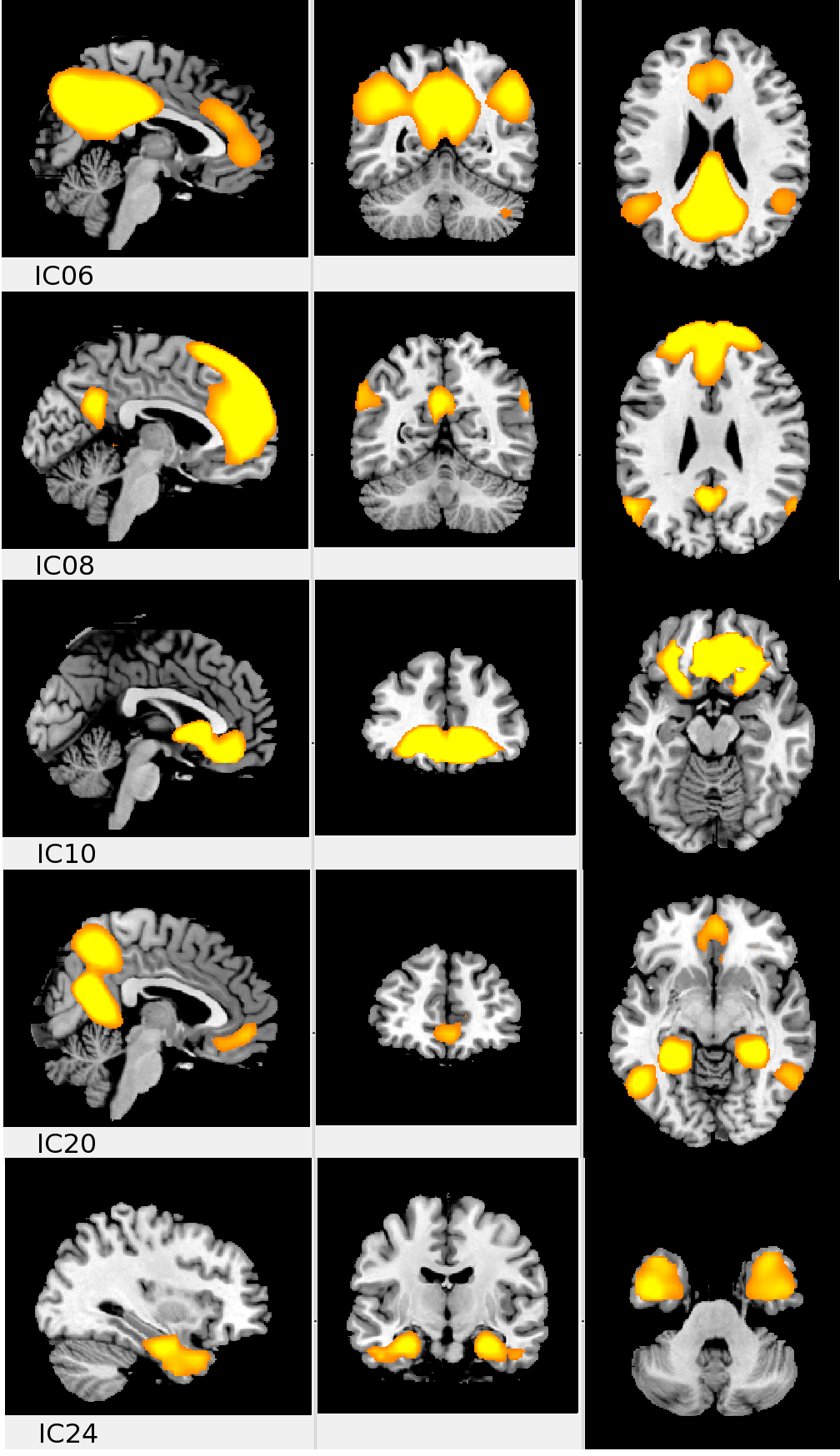

Supplement: Additional file 1: — Supplementary Methods and Results. [file 12888_2015_457_MOESM1_ESM.docx]
